# Decreased cortical FADD protein is associated with clinical dementia and cognitive decline in an elderly community sample

**DOI:** 10.1186/s13024-017-0168-x

**Published:** 2017-03-20

**Authors:** Alfredo Ramos-Miguel, Jesús A. García-Sevilla, Alasdair M. Barr, Thomas A. Bayer, Peter Falkai, Sue E. Leurgans, Julie A. Schneider, David A. Bennett, William G. Honer, M. Julia García-Fuster

**Affiliations:** 1 BC Mental Health and Addictions Research Institute, Vancouver, Canada; 2 Department of Psychiatry, University of British Columbia, Vancouver, Canada; 3 IUNICS, University of the Balearic Islands, Ctra. de Valldemossa km 7.5, E-07122 Palma de Mallorca, Spain; 4 Instituto de Investigación Sanitaria de Baleares, Palma de Mallorca, Spain; 5 Department of Anesthesiology, Pharmacology and Therapeutics, University of British Columbia, Vancouver, Canada; 6 Department of Psychiatry, University Medicine Goettingen, Goettingen, Germany; 7 Department of Psychiatry and Psychotherapy, Ludwig-Maximilians-University Munich, Munich, Germany; 8 Rush Alzheimer’s Disease Center, Rush University Medical Center, Chicago, USA

**Keywords:** Alzheimer’s disease, Aging, Neurotoxicity, Neuroplasticity, Apoptosis

## Abstract

**Background:**

FADD (Fas-associated death domain) adaptor is a crucial protein involved in the induction of cell death but also mediates non-apoptotic actions via a phosphorylated form (p-Ser194-FADD). This study investigated the possible association of FADD forms with age-related neuropathologies, cognitive function, and the odds of dementia in an elderly community sample.

**Methods:**

FADD forms were quantified by western blot analysis in dorsolateral prefrontal cortex (DLPFC) samples from a large cohort of participants in a community-based aging study (Memory and Aging Project, MAP), experiencing no-(NCI, *n* = 51) or mild-(MCI, *n* = 42) cognitive impairment, or dementia (*n* = 57).

**Results:**

Cortical FADD was lower in subjects with dementia and lower FADD was associated with a greater load of amyloid-β pathology, fewer presynaptic terminal markers, poorer cognitive function and increased odds of dementia. Together with the observations of FADD redistribution into tangles and dystrophic neurites within plaques in Alzheimer’s disease brains, and its reduction in APP23 mouse cortex, the results suggest this multifunctional protein might participate in the mechanisms linking amyloid and tau pathologies during the course of the illness.

**Conclusions:**

The present data suggests FADD as a putative biomarker for pathological processes associated with the course of clinical dementia.

**Electronic supplementary material:**

The online version of this article (doi:10.1186/s13024-017-0168-x) contains supplementary material, which is available to authorized users.

## Background

Aging is a relevant risk factor for Alzheimer’s disease (AD) [1], which is the main cause of dementia and is characterized by deposition of amyloid β in neuritic plaques, accumulation of tau in intracellular neurofibrillary tangles, and neuronal loss (see review in [2]). Cognitive decline is a prerequisite for the clinical diagnosis of dementia associated with AD, and usually correlates better with neurofibrillary tangles of hyperphosphorylated tau than with amyloid β plaques (reviewed in [3, 4]). Although nearly all brains in old age contain some pathological signs of AD, only some individuals develop the disease [5–9], which seems to be greatly influenced by differences in cognitive reserve [10–12]. This premise, together with the time-course of the pathophysiology of the disease (i.e., preclinical stage initiated 15–20 years prior to emergence of clinical signs; [4]) suggests that these pathological markers may not be sufficient or necessary to initiate cognitive decline in humans. Consequently, the identification of new biomarkers for diagnosis and for a sensitive assessment of the progression of AD (i.e., cognitive decline) is an important area of current research.

In line with this, cell death mediated via apoptosis has been thought to be one of the possible underlying mechanisms of neuronal cell loss in AD (see review in [13]). In fact, several studies have been performed in postmortem brain tissue from AD patients on some of the cellular components of the apoptotic pathway [13]. For example, the expression of some components of the extrinsic pathway, like the cell death receptor Fas (e.g., [14–16]), its ligand Fas-L [17] and some effector caspases [18, 19] showed evidence of apoptosis (i.e., DNA fragmentation determined by TUNEL method, [18]) in the brains of AD patients. However, several contradictory results have also been described, suggesting that the apoptotic mechanism of neuronal death in AD might be infrequent or undetectable (e.g., [20, 21]), and that in fact, even cell survival mechanisms might take place as an adaptive response to a prior insult (see revision in [13]).

In terms of cell fate regulation (i.e., balance between cell death and survival), a key adaptor molecule of the apoptotic Fas receptor [22], the FADD (Fas-associated death domain) is a crucial protein involved in the induction of cell death but also able to mediate non-apoptotic actions (cell survival, differentiation and neuroplasticity) via a phosphorylated form (p-Ser194-FADD) and its nuclear localization [23, 24]. Multifunctional FADD works in vivo as a common and major signaling step in the initial activation of structurally different receptors (e.g. neurotransmitter G protein-coupled receptors and receptor channels; see [25–27]). Although FADD has a crucial role during embryogenesis/development [28], little is known about its expression or functions as the brain ages [29] and in age-related neurophatologies. In AD one prior study showed that although FADD signaling pathway was triggered within the basal forebrain cholinergic neurons, as FADD-positive tangle-like structures were localized in neurons, there was no apoptotic cell death as measured by DNA fragmentation [30]. Another study showed that the induction of neural apoptosis produced by amyloid β in hippocampal neuron cultures was mediated via FADD and caspase-8 activity [31]. However, none of these studies explored the potential link between brain FADD (or other apoptotic markers) variations and cognitive decline or AD-related pathology.

Against this background, this study investigated the possible associations of FADD forms (i.e., speculated increase of pro-apoptotic FADD) with the presence and severity of multiple age-related neuropathologies, as well as with cognitive function and the risk of clinical dementia in an elderly large community sample. Cortical expression of FADD forms (i.e., pro-apoptotic FADD and anti-apoptotic p-FADD) were measured in postmortem tissue samples from a large cohort of community-dwelling participants of the Memory and Aging Project (MAP) [32], with or without clinical diagnosis of dementia, and representative of the broad range of cognitive impairment in the overall elderly, aging population. Moreover, to further ascertain the possible role of FADD regulation in AD, we utilized APP23 transgenic mice, which overexpress mutant human amyloid precursor protein (APP) and develop brain amyloid β deposits in brain progressively with age [33]. A preliminary report of a portion of this work was presented at the 55th Annual Meeting of the American College of Neuropsychopharmacology [34].

## Methods

### Selection of MAP participants: cognitive and neuropathological evaluations

The Memory and Aging Project (MAP) recruits elderly volunteers (more than 1,800 since 1997) without known dementia at enrollment, living in the metropolitan area of Chicago (IL, USA) [9, 32]. All participants signed an informed consent and an Anatomic Gift Act for organ donation upon death. The Institutional Review Board of Rush University Medical Center approved this study. The overall follow-up rate was 95% and the autopsy rate exceeded 80% resulting in more than 650 autopsies. A large number of MAP participants (*n* = 426) from consecutive autopsies were used in a recent study (see [35]). From those, *n* = 150 participants were pseudorandomly selected using a random sampling tool in JMP software (version 12.1; SAS Institute, NC, USA), and included in the present study. See Table 1 for a summary of their demographic, cognitive (no cognitive impairment, NCI; mild cognitive impairment, MCI; clinical dementia) and pathological characteristics. Apolipoprotein E (*APOE*) genotyping was performed with PCR assays by Agencourt (Beckman Coulter Genomics, Brea, CA, USA).

**Table 1:** Demographic, cognitive and pathological characteristics^a^ of MAP participants included in the present study

Variable	All participants (*n* = 150)	NCI (*n* = 51)	MCI (*n* = 42)	Dementia (*n* = 57)
Demographic				
Female, no. (%)	105 (70%)	40 (78%)	27 (64%)	38 (67%)
Age at death, years	88.7 ± 6.3	86.7 ± 6.7	88.1 ± 6.8	90.9 ± 4.7
Education, years	14.6 ± 2.9	13.9 ± 2.5	15.0 ± 2.8	15.0 ± 3.3
Race, no. W:AA	146:4	49:2	41:1	56:1
*APOE* ε4 carrier, no. (%)	33 (22%)	7 (14%)	11 (26%)	15 (26%)
PMI, hours	6.6 ± 3.8	6.2 ± 2.1	7.6 ± 5.6	6.2 ± 3.1
Cognitive function proximate to death				
Global cognition score	−0.77 ± 1.01	0.10 ± 0.39	−0.47 ± 0.43	−1.80 ± 0.78
MMSE	21.9 ± 8.2	27.7 ± 1.9	24.9 ± 4.6	14.4 ± 8.1
Pathological				
Macroinfarcts, no (%)^b^	45 (30%)	10 (20%)	15 (36%)	20 (35%)
Lewy bodies, no (%)^b^	25 (17%)	4 (8%)	1 (2%)	20 (35%)
Hippocampal sclerosis, no (%)^b^	14 (9%)	0 (0%)	4 (10%)	10 (18%)
Arteriolosclerosis^b^	1.39 ± 0.87	1.24 ± 0.84	1.38 ± 0.82	1.53 ± 0.93
Amyloid plaques^c^	5.3 ± 5.9	2.9 ± 3.3	5.5 ± 6.5	7.3 ± 6.5
Tangles^c^	1.1 ± 3.0	0.4 ± 1.7	1.1 ± 3.0	1.8 ± 3.7

*Abbreviations*: *AA* Afro-American, *AD* Alzheimer’s disease, *MAP* Memory and Aging Project, *MCI* mild cognitive impairment, *MMSE* mini mental state examination, *NCI*, no cognitive impairment, *PMI* postmortem interval, *SD* standard deviation, *W* White

^a^Values are mean ± SD unless noted otherwise

^b^Global values

^c^Values obtained in the contralateral dorsolateral prefrontal cortex (DLPFC) by immunohistochemistry with specific antibodies

Prior reports extensively reported the methodological approaches to perform systematic cognitive, clinical and neuropathological evaluations [9, 36]. Annual cognitive evaluations included a series of 21 standard tests, 19 of which were used for summary measures of episodic, semantic and working memory, perceptual speed, and visuospatial ability, and finally summarized into one single variable to derive a global cognitive function score [9, 37]. The mini mental state examination (MMSE) is also reported for comparison to other studies (see Table 1). A board-certified neuropsychologist blind to all pathological data reviewed test results and rated the level of cognitive impairment. A study clinician evaluated each participant and diagnosed dementia and AD following the National Institute of Neurological and Communicative Disorders and Stroke and the Alzheimer’s Disease and Related Disorders Association criteria [38] implemented as described [39]. Cognitive impairment not meeting the criteria for dementia was diagnosed as mild cognitive impairment (MCI) as described [40]. NCI refers to those without MCI or dementia [41].

The pathological examinations were made by a board-certified neuropathologist, blind to all clinical data. AD pathology (i.e. plaques and tangles) was evaluated in formalin-fixed, paraffin-embedded sections from multiple key brain regions in the frontal, temporal, parietal, and occipital lobes, as previously described [42], although only data from the dorsolateral prefrontal cortex (DLPFC) was used for statistical modeling, unless otherwise specified. Briefly, sections from all subjects and brain areas were assessed using both a modified Bielschowsky silver staining for counts of diffuse and neuritic plaques, and neurofibrillary tangle (NFTs), as described [43]. Immunocytochemistry with amyloid-β (clones 10D5 or 4G8) to quantify the percent area occupied by amyloid-β by image analysis, and phosphotau (clone AT8) antibodies – to quantify the density of tau tangles by stereology [42]. The severity and/or stage of AD in each participant was later addressed following the National Institute on Aging (NIA)-Reagan criteria, which incorporates the Consortium to Establish a Registry of Alzheimer’s Disease (CERAD) scale [44], and Braak staging [45]. Other neuropathologies, including cerebrovascular diseases (macroscopic and microscopic infarcts, arteriolosclerosis and atherosclerosis), Lewy bodies, and hippocampal sclerosis, were also examined as described elsewhere [9, 36]. Stereological approaches to account for resting, activated or total microglial cells in the DLPFC were detailed earlier [46].

### Animals

APP23 transgenic mice, overexpressing a variant of human APP carrying the ‘Swedish double mutation’ KM670/671NL [33], and wild-type (WT) littermates were provided by Novartis Pharma (Basel, Switzerland) at different ages (3, 12 and 22 months old; *n* = 5–6 per genotype and age). Mice were killed by decapitation, and the frontal cortex was dissected and prepared for further Western blot (WB) analysis [47].

### Tissue collection, immunoassays and quantification of target proteins

At the time of autopsy of MAP participants, tissue slabs from the middle-frontal gyrus (Brodmann’s area 46/9) of the DLPFC were dissected following a standard human brain atlas [48], and stored at −80 °C. The DLPFC was selected for its central role in complex cognitive tasks and contribution to age-related cognitive decline [49]. For further immunoassays, grey matter tissue was carefully sampled from each of the slabs avoiding thawing. Total homogenates from DLPFC samples (40–80 mg) were prepared in ice-cold PBS pH 7.4 following usual procedures [11]. Then, protein concentrations were determined by DC assay (Bio-Rad, Hercules, CA, USA) and samples were adjusted to equal concentrations with homogenization buffer. A greater amount of a reference MAP cortical sample (~1 g) was homogenized and prepared following the same conditions to be used as an internal control (i.e., standard sample) in the immunoblot assays.

Cortical samples from MAP participants (40 μg) or APP23/WT mice (10 μg) were resolved by electrophoresis on 10% SDS–PAGE minigels (Bio-Rad Laboratories, Hercules, CA, USA). Every gel was run with 14 brain samples, including 11 MAP participants (or 11 APP23/WT mice samples) selected randomly, and the triplicate standard samples (the reference MAP sample or a pool of WT mice) space-loaded across the gel, and a molecular weight ladder (Bio-Rad). Each cortical sample was assessed at least three times in different gels (i.e., with randomly allocated brain samples) on different days. Following electrophoretic separation, proteins were transferred to nitrocellulose membranes, then incubated overnight at 4 °C in blocking solution with anti-FADD (H-181) (1:5000; Santa Cruz Biotechnology, CA, USA) (see [50, 51] for labeling in post-mortem human brain tissue). The secondary antibody was incubated for 1 h at room temperature (1:5000 dilution; Cell Signaling). Immunoreactivity of target proteins was detected with ECL reagents (Amersham, Buckinghamshire, UK) and signal of bound antibody was visualized by exposure to autoradiographic film (Amersham ECL Hyperfilm) for 1 to 60 min, then quantified by densitometric scanning (GS-800 Imaging Calibrated Densitometer, Bio-Rad). Quantification of p-FADD and ß-actin protein contents were performed by sequentially stripping and reprobing all membranes, first for anti-phospho-Ser194 FADD (1:1000; Santa Cruz Biotechnology, CA, USA), and then for anti-ß-actin (clone AC-15) (1:10000; Sigma-Aldrich, MO, USA). For each sample, immunoreactivity of FADD or p-FADD was first divided by that of ß-actin (i.e., protein content data normalization) within the same gel, and then calculated as a percentage of in-gel standards. The mean value for each sample from at least three separate gels was used as a final estimate. During the above procedures, the experimenter was blind to the demographic, cognitive and pathological characteristics of MAP participants.

Quantification of the presynaptic proteins syntaxin-1, synaptosomal-associated protein of 25 kDa (SNAP25), vesicle-associated membrane protein (VAMP) and syntaxin-binding protein-1 (STXBP1) was performed previously by either enzyme-linked immunosorbent assay [11] or Western blotting [35], in the same brain samples. The DLPFC or overall brain immunodensities of the three SNARE proteins (i.e. syntaxin-1, SNAP25, VAMP) were z-scored and averaged to obtain a variable accounting for cortical or global synapse density, directly related to synaptopathy [52].

### Immunofluorescence analysis

In an attempt to better characterize the potential relationship between FADD (i.e., cellular and anatomical localization), cognitive decline and AD pathology, 6 MAP subjects (3 pathology-free NCI subjects and 3 dementia cases with confirmed cortical AD pathology) were selected for further immunofluorescence analysis. Tissue blocks from the DLPFC (BA9) were cut coronally with a vibrating microtome (Leica, Nussloch, Germany) to a thickness of 40 μm, and floating sections were cryo-preserved in solution at −20 °C until further use. Antigen retrieval was done in 20 mM citrate buffer (pH 6.0, 80 °C, 20 min) and was followed by blocking sections and overnight incubation at 4 °C with one of the following primary antibodies: anti-FADD (H-181, Santa Cruz, 1:50), anti-NeuN (clone A60) (1:250; Chemicon, CA, USA), anti-syntaxin-1 (clone SP7) (1:1000; [53]), anti-amyloid β (clone 6 F/3D) (1:100; Dako, Glostrup, Denmark), anti-mis-folded pathologic tau (clone Alz-50) (1:500; [54]). The next day, sections were incubated with Alexa Fluor® 488-, 555- or 647-conjugated anti-mouse or anti-rabbit secondary antibodies (1:500; Southern Biotech, AL, USA). Auto-fluorescence was eliminated by incubation in 0.1% Sudan Black B and 70% ethanol solution for 15 min. Sections were mounted in gelatin-coated slides and protected with an anti-fade mounting reagent. A series of orthogonal images were captured at a resolution of 1024×1024 pixels using a LSM 5 Pascal confocal microscope (Zeiss; Jena, Germany) and were visualized using a 63x/1.2 N.A. water-immersion objective. Co-staining of FADD with NeuN and syntaxin-1 was assessed to determine presence in neurons and/or synapses, while co-staining of FADD with amyloid β or mis-folded pathologic tau determined its presence within plaques and/or tangles, respectively.

### Statistical analyses

Data were analyzed and plotted with GraphPad Prism, Version 6 (GraphPad Software, CA, USA) and/or JMP. The level of significance was *p* ≤ 0.05. Initial comparisons of the cortical pathologic burden between clinically diagnosed groups were done by Kruskal-Wallis followed by Dunn’s test. Following WB assays, FADD and p-FADD immunodensities were normalized to corresponding β-actin values, and calculated as a percentage of in-gel standards (see [35]). While these values were used for plots, later statistical modeling procedures required a logarithmic transformation and standardization in order to obtain normally distributed measures, as confirmed by Shapiro-Wilk test. Multivariate analyses were performed to detect potentially confounding factors (e.g. demographic variables, *APOE* genotype, tobacco or alcohol consumption, psychotropic drug prescription, etc.) influencing cortical FADD/p-FADD levels, as well as other interesting associations of these molecules with multiple clinical, pathologic or neurochemical variables measured along the study. Among the confounding factors, only postmortem interval (PMI) significantly correlated with FADD (*r* = −0.278; *p* < 0.001) and p-FADD (*r* = 0.240; *p* < 0.003) brain values, and therefore was included as a covariate (among other variables) in all follow-up models. Differences in FADD and p-FADD immunodensities between clinically diagnosed (i.e., NCI, MCI, dementia) or pathologically graded (i.e. CERAD scaled or Braak staged) groups were assessed by analysis of covariance (ANCOVA), controlling for sex, age, years of education and PMI, followed by Tukey’s HSD test. Given the potential effects of cortical FADD levels on cognitive performance, logistic or linear regression models (controlled for sex, age, education, PMI, and *APOE* genotype) were evaluated with clinical dementia or cognitive function proximal to death as respective outcomes, and pathological and neurochemical variables as predictors (see [35]). Additionally, we constructed univariate random-effects models, controlled for demographics and neuropathologies as above, to study the potential influence of FADD cortical immunodensites (measured postmortem) on the cognitive decline rates of MAP participants, as previously described [12]. Note that these models assume fixed values of cortical FADD levels longitudinally, a limitation that must be considered when interpreting the results.

For WB experiments involving transgenic mice, data was analyzed with two-way ANOVA, in which genotype (WT vs. APP23) and age (3, 12 and 22 months old) were treated as independent variables, followed by multiple t-tests for two-group comparisons at each age.

ImageJ 2.0 (NIH, MA, USA) was used to determine and quantify the extent of colocalization between two immunofluorescent dyes in confocal imaging using an unbiased built-in method [55, 56].

## Results

### Characteristics of MAP participants

**Fig. 1:**
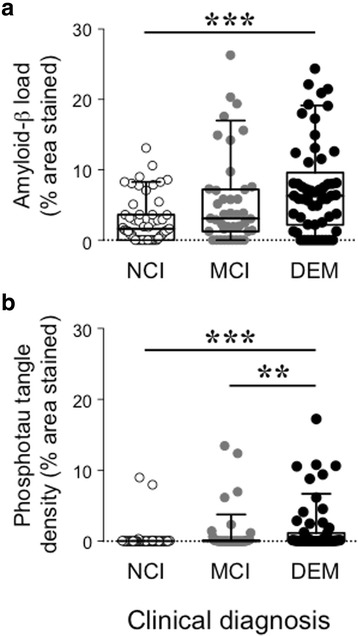
Measures of Alzheimer’s disease pathology in the DLPFC of MAP participants in relation to their clinical diagnoses. Following standard immunohistochemical assays with antibodies against (**a**) amyloid β (clones 10D5 or 4G8) and (**b**) phosphotau (clone AT8), the acquired images were thresholded and the percent area immunostained was estimated for each participant and plotted by clinical diagnosis criteria into no cognitive impairment (NCI, *n* = 51), mild-cognitive impairment (NCI, *n* = 42) or dementia (DEM, *n* = 57). Whiskers represent 10th and 90th percentiles and boxes enclose interquartile ranges crossed by the median of AD pathology scores within groups. Differences among groups were assessed by Kruskal-Wallis followed by Dunn’s multiple comparison test. **p < 0.01 and ***p < 0.001

Descriptive statistics for demographic, cognitive and pathological characteristics of MAP participants are summarized in Table 1. Out of the total of 150 MAP participants, 51 subjects presented no cognitive impairment (NCI), 42 displayed mild cognitive impairment (MCI), while 57 were clinically diagnosed with dementia (see Table 1, cognitive function proximate to death). As expected, common AD disease pathology (i.e. amyloid-β load and tau tangle density) in the DLPFC was more abundant in dementia cases, as compared to NCI participants (2.5–5.1 fold, *p* < 0.001) (see Fig. 1). None of these markers could separate MCI from NCI participants, while dementia cases showed greater density of phosphotau tangles than MCI participants (1.6 fold, *p* = 0.003) (Fig. 1b). Notably, and as previously reported in larger epidemiologic studies [7, 9] there is ample variability in all clinically diagnosed groups.

### Association of cortical FADD with common age-related pathology

The total amounts of FADD protein forms (FADD and p-FADD) were quantified in the DLPFC of MAP participants and normalized by β–actin protein content. Our research group, which has been working with these specific antibodies for over a decade, characterized FADD and p-FADD specific bands with several antibodies and enzymatic dephosphorylation assays in brain tissue [50, 51, 57, 58] (see detailed revisions on these antibodies characterization in [59, 60]). As reviewed in [60], and shown in the present results, total FADD is mainly immunodetected as a 51-kDa dimeric form, while p-FADD is immunodetected as a 116-kDa oligomeric form both in rodent and human brains. The results revealed large variability among MAP participants for FADD immunodensities (interquartile range = 20–83%), and p-FADD (57–102%) as compared to that of β-actin (93–105%).

**Fig. 2:**
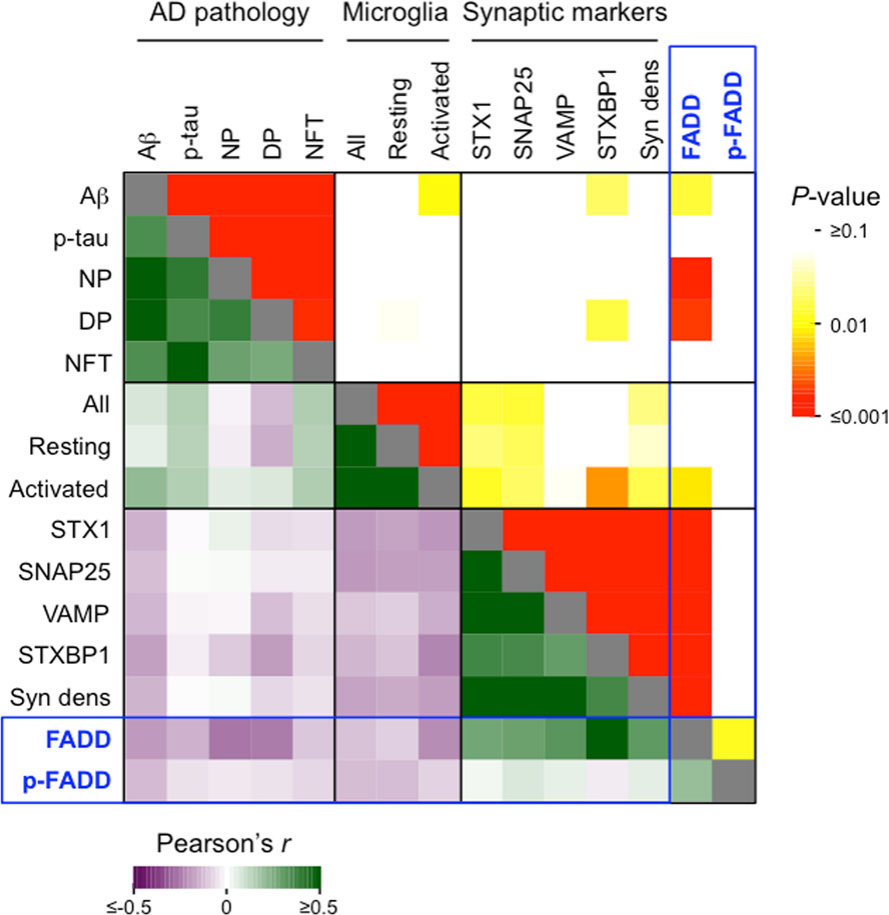
Heatmap of Pearson’s *r-* (bottom-left) and *P*- (top-right) values following multiple pairwise correlations between the pathologic, stereological and neurochemical variables indicated. Abbreviations: Aβ, amyloid β; AD, Alzheimer’s disease; DP, diffuse plaques; NFT, neurofibrillary tangles; NP, neuritic plaques; p-, phospho-; SNAP25, synaptosomal-associated protein of 25 kDa; STX1, syntaxin-1; STXBP1, syntaxin-binding protein-1; Syn dens, synaptic density; VAMP, vesicle-associated membrane protein

**Fig. 3:**
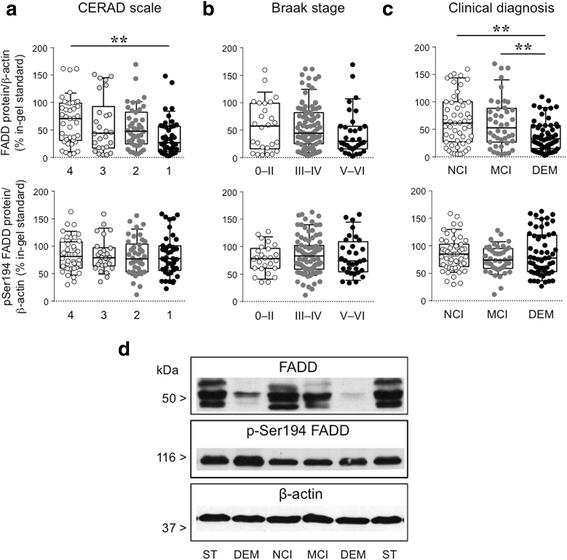
Regulation of FADD (upper plots) and p-FADD (bottom plots) protein forms in the DLPFC of MAP participants ranked either by: **a** CERAD severity scale according to plaque pathology, and displaying no (4, *n* = 40), sparse (3, *n* = 26), moderate (2, *n* = 42), or frequent (1, *n* = 42) plaque load; **b** Braak staging according to the spread of the tauopathy, and displaying no or transentorhinal deposition (0–II, *n* = 26), limbic spread (III–IV, *n* = 92), or neocortical spread (V–VI, *n* = 32); or (**c**) clinical diagnoses with no cognitive impairment (NCI, *n* = 51), mild-cognitive impairment (NCI, *n* = 42) or dementia (DEM, *n* = 57). Whiskers represent 10th and 90th percentiles of FADD or p-FADD values (normalized by β-actin protein content), with boxed interquartile ranges crossed by the median for each experimental group and expressed as percentage of an in-gel-standard. Differences among groups were assessed (after log-transformation and standardization of the datasets) by ANCOVA controlling for age, sex, education and PMI followed by Tukey’s HSD *post hoc* test. **p < 0.01. **d** Representative immunoblots of FADD, p-FADD and β-actin, with various participants and standard (ST) samples. The molecular masses of the various proteins are indicated in kDa. Full gel images are included in Additional file 2: Figure S2

Protein expression levels of FADD species did not differ in the DLPFC of MAP participants displaying cerebrovascular diseases (including infarcts), Lewy bodies, cortical atrophy, or hippocampal sclerosis from those who did not (data not shown). By contrast, FADD levels (but not those of p-FADD) negatively correlated with cortical amyloid-β load (*r* = −0.197; *p* = 0.038), without apparent association with phosphotau density (*r* = −0.124; *p* = 0.118) (Fig. 2). The association between FADD and plaque pathology was stronger when using neuritic (*r* = −0.266; *p* < 0.001) and diffuse (*r* = −0.258; *p* = 0.006) plaque counts on the silver-stained sections, rather than amyloid-β immunodensities, which are more sensitive (Fig. 2). Consequently, subjects graded with definite AD pathology by CERAD had lower cortical FADD (but not p-FADD) immunodensities (−45%, *p* = 0.004) compared to plaque pathology-free participants (Fig. 3a). These differences in FADD contents were not found in association with Braak stage (Fig. 3b). Of note, the above neuropathological assessments were part of previous studies, where detailed information on image processing and quantitative approaches was reported [36, 41–43].

Additionally, greater FADD cortical immunodensity was associated with lower number of activated (*r* = −0.224; *p* = 0.018, *n* = 110), but not resting or total, microglial cells (Fig. 2), as characterized and quantified previously [46]. Confocal microscopy studies labeling FADD and HLA-DR-positive (activated) microglia revealed significant colocalization between both markers within microglial processes, but not in their nuclei (Additional file 1: Figure S1). This type of overlap might be attributed to the engulfment of neuronal-derived apoptotic material, and therefore activated microglia might mediate pro-apoptotic FADD clearance. By contrast, higher FADD immunodendities, but not p-FADD, were strongly associated with greater amounts of most presynaptic markers quantified in the same cortical samples in prior studies [11, 35], including syntaxin-1 (*r* = 0.274; *p* = 0.004), synaptosomal-associated protein of 25 kDa (SNAP-25; *r* = 0.290; *p* = 0.002), vesicle-associated membrane protein (VAMP; *r* = 0.327; *p* < 0.001), and syntaxin-binding protein-1 (STXBP1; *r* = 0.520; *p* < 0.001) (Fig. 2). Of note, loss of these markers is related to synaptic pathology in aging and AD [61]. An index of synapse density was estimated by averaging syntaxin-1, SNAP-25 and VAMP immunodensities (i.e., the so-called SNARE proteins) in order to obtain a variable accounting for cortical synaptopathy in MAP participants in later statistical models. As expected, this index also correlated with FADD values (*r* = 0.312; *p* < 0.001).

### Association of cortical FADD with clinical dementia and cognitive function

Comparing MAP participants by clinical diagnoses revealed that FADD was lower in the DLPFC of subjects with dementia relative to NCI (−42%, *p* = 0.003) and MCI (−27%, *p* = 0.006), while p-FADD was not different (Fig. 3c,d). Given these associations, we addressed the hypothesis that lower cortical FADD levels may contribute to increased likelihood of dementia and/or greater cognitive impairment, either by mediating the effects of age-related neuropathologies or independent of these indices. We therefore performed a series of logistic and linear regression models taking into account demographics, multiple age-related pathologies (i.e., amyloid plaques, tangles, Lewy bodies, cerebrovascular diseases, hippocampal sclerosis), and overall synaptic density, omitting or including cortical FADD levels in the models (see Table 2 and data not shown).

**Table 2:** Regression models showing the associations of FADD levels in the DLPFC of MAP participants (and relevant covariates) with clinical dementia and cognitive function

	Clinical dementia^a^			Cognitive function^b^		
Model terms	Odds ratio	95% CI	*P*-value	Estimate	SD	*P*-value
Age at death	1.104	1.025–1.197	0.0117*	−0.0293	0.0118	0.0144*
Sex	1.231	0.440-3.433	0.6887	0.0856	0.0818	0.2976
Education years	1.133	0.966–1.339	0.1319	−0.0278	0.0260	0.2877
PMI	0.928	0.804–1.039	0.2401	−0.0052	0.0203	0.7991
*APOE* ε4 allele	0.752	0.235–2.267	0.6195	0.0369	0.0921	0.6892
Macroinfarcts^c^	1.295	0.498–3.328	0.5913	−0.1040	0.1616	0.5208
Lewy bodies^c^	9.190	2.833–35.790	0.0005*	−0.5993	0.1971	0.0028*
Hipp. sclerosis	3.935	0.832–21.845	0.0930	−0.1160	0.2813	0.6808
Arteriolosclerosis^c^	1.271	0.783–2.090	0.3350	−0.1022	0.0824	0.2166
Amyloid plaques^d^	1.366	0.974–1.948	0.0756	−0.1186	0.0588	0.0458*
Tangles^d^	1.040	0.906–1.208	0.5865	−0.0887	0.0265	0.0011*
Synaptic density^e^	1.798	0.895–3.891	0.1151	−0.1003	0.1065	0.3475
FADD^f^	0.433	0.248–0.717	0.0019*	0.4993	0.1840	0.0075*

*Abbreviations*: *C.I.* confidence intervals, *DLPFC* dorsolateral prefrontal cortex, *FADD* Fas-associated protein with death domain, *Hipp.*, hippocampal, *MAP* Memory and Aging Project, *MCI* mild cognitive impairment, *NCI* no cognitive impairment, *PMI* postmortem interval, *S.D.*, standard deviation, *SNAP-25*, synaptosomal-associated protein of 25 kDa, *VAMP*, vesicle-associated membrane protein

^a^Logistic regression model of the estimated odds ratios of clinical dementia vs. non-dementia (i.e. NCI and MCI participants) per unit of regressor

^b^Linear regression model predicting global cognitive function nearest to death

^c^Global values

^d^Values obtained in the contralateral DLPFC by immunohistochemistry with specific antibodies

^e^Estimated as the mean value of calculated densities of the presynaptic proteins syntaxin-1, SNAP-25 and VAMP in the same brain samples

^f^Values normalized by β-actin

*Statistically significant *P*-value < 0.05

As expected, age and the presence of Lewy bodies were associated with both higher odds of dementia and poorer cognitive performance prior to death. Surprisingly, in the present MAP subset, cerebrovascular diseases and hippocampal sclerosis were related with neither dementia nor cognitive function. However, in previous studies using a much larger MAP sample, these latter associations were indeed observed (see e.g. [12]). Interestingly, the effect of DLPFC amyloid-β load on the likelihood of dementia, which was marginally significant when the FADD data was not included in the model (data not shown), was not significant after addition of FADD suggesting a mediation effect. Thus, participants with lower FADD cortical density displayed a significantly greater likelihood of dementia (odds ratio = 0.433, *p* = 0.002). For example, a MAP participant with average demographic and pathologic characteristics had a 2.5 fold-higher likelihood of dementia if the cortical density of FADD is in the lower quartile versus the higher quartile. Likewise, higher FADD levels in the DLPFC were associated with better global cognitive function in MAP participants (*β* = 0.499, *p* = 0.008), while the DLPFC load of amyloid-β showed a marginal but significant effect on cognitive function (*β* = −0.119, *p* = 0.046) after adding FADD to the linear regression model. Cortical FADD immunodensity did not mediate the large effects of the DLPFC tauopathy on cognitive function; and surprisingly had no impact on the risk of clinical dementia in the current MAP sample (Table 2). Importantly, after controlling for the demographic and pathologic indices in Table 2, variations in DLPFC FADD immunodensity explained 3.44% of the total variance in cognition among MAP participants. Of note, the associations between the clinical outcomes and the DLPFC pathologic and neurochemical variables reported in Table 2 were very similar to those observed when using the overall brain measurements of amyloid and tau pathologies (data not shown).

**Fig. 4:**
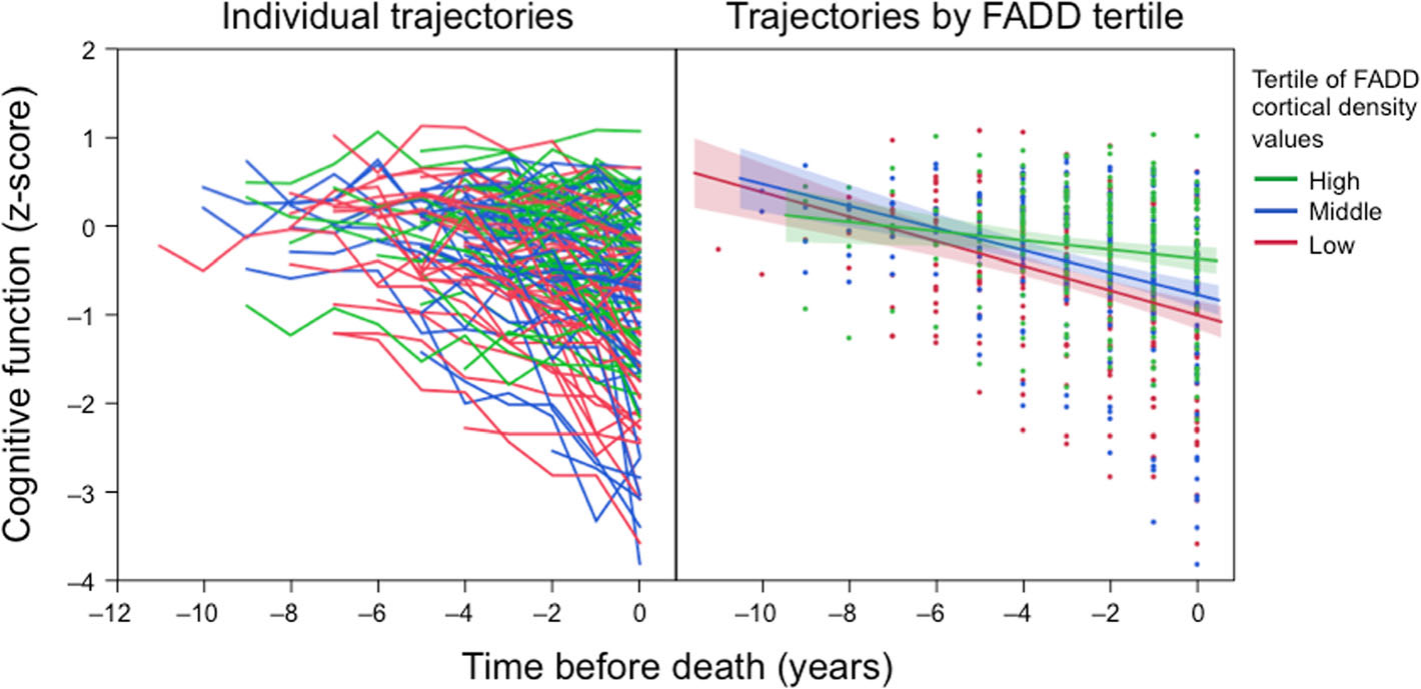
Cognitive decline trajectories of MAP participants ranked by cortical FADD levels. Global cognitive function was evaluated annually after enrolment, for 5.6 years in average (range 3–12 years), and the scores were standardized as described in Methods. Participants were then aligned longitudinally by the last cognitive test before death. Participants were divided into groups according to their tertile of FADD cortical expression levels. The spaghetti plot on the left represents individual cognitive trajectories across the study. The panel on the right represents individual annual scores (points), with the best fit (solid line) and the 95% confidence interval (shaded area) of the cognitive trajectories for each FADD-ranked group overlapped

Given that clinical diagnoses were not performed longitudinally, diagnoses of NCI/MCI/dementia were based on cognitive evaluations nearest death. Therefore, establishing an association between the present neurochemical data and the duration of the illness was not possible. Because the onset of cognitive decline was somewhat variable (see left panel in the Fig. 4), we performed random-effects models to evaluate the potential association between longitudinally ascertained cognitive decline rates and postmortem cortical immunodensities of FADD, adjusting for demographics and neuropathologies. Although a trend of faster cognitive decline rate was observed for those subjects within the lower tertile of FADD cortical values (see Fig. 4), in the statistical approaches using univariate random-effects models (controlling for all above confounders and pathologies) this neurochemical variable only showed a marginal, non-significant association with the annually evaluated cognitive function (*β* = 0.266, *p* = 0.080), possibly because the current sample size is underpowered for this type of analysis.

### Differences in FADD cortical distribution in clinical dementia

**Fig. 5:**
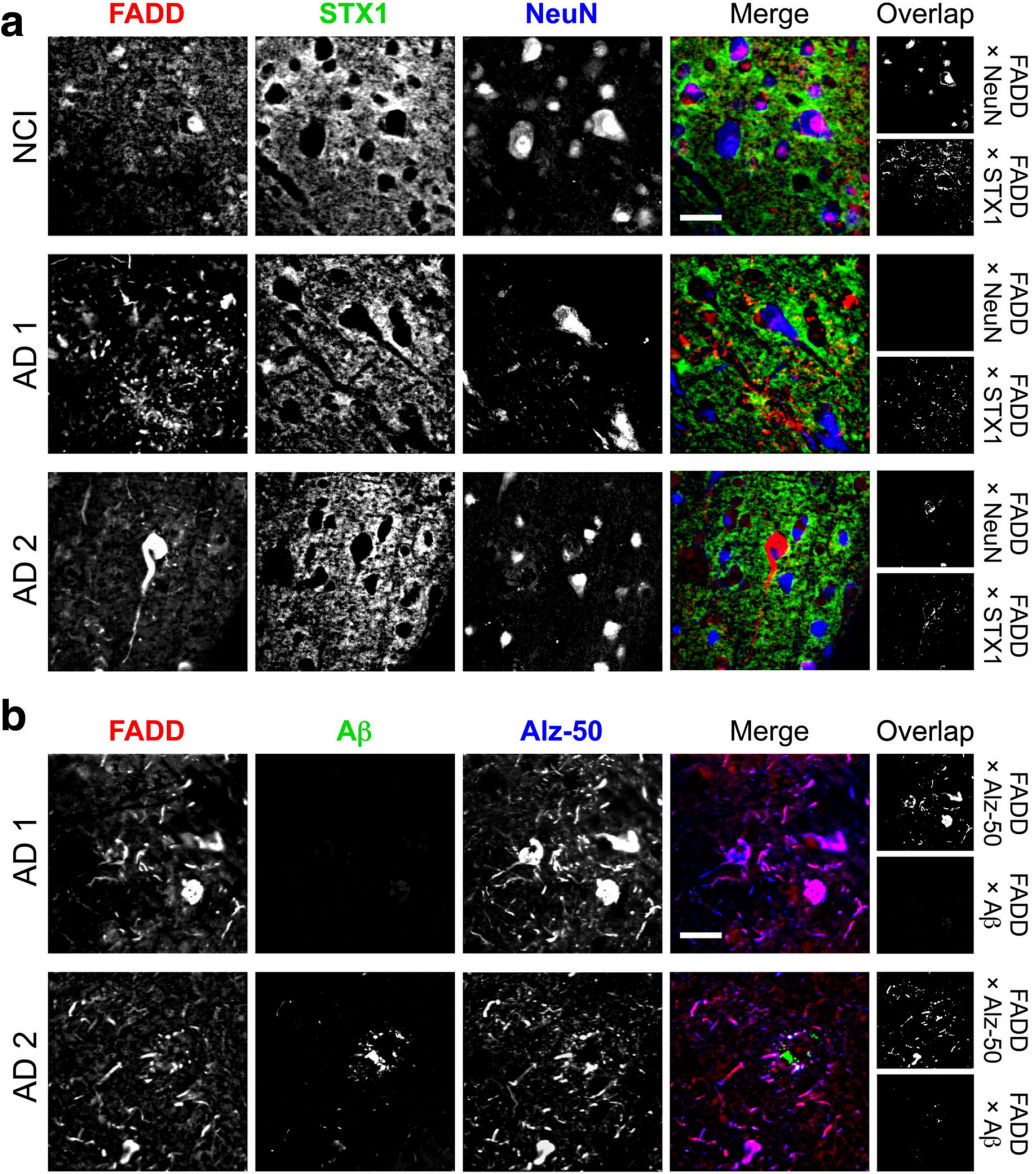
Immunofluorecence characterization of FADD in the DLPFC of neuropathology-free NCI (*n* = 3) and Alzheimer’s diseased (AD; *n* = 3; two different subjects shown) MAP participants. Single-channel (in greys) or merged (in RGB) confocal images correspond to triple co-immunolabeled sections with antibodies against FADD (H181, Santa-Cruz, 1:50) combined with either (**a**) synatxin-1 (STX1, clone SP7, locally produced, 1:1000) and NeuN (Chemicon, clone A60, 1:250), or (**b**) beta-amyloid (Aβ, clone 6 F/3D, Dako, 1:100) and misfolded, pathologic tau (clone Alz-50, locally produced, 1:500). In merged images, colors were arbitrarily assigned (as indicated at the top) to maximize overlap visualization. Overlap panels on the right are ImageJ-generated bitmaps highlighting those pixels where significant colocalization over an unbiased threshold of intensities between the indicated channels were detected in the corresponding pairwise colocalization analyses. Note the change in FADD localization in NCI (mainly in neuronal nuclei and soma, and also some neuropil staining) compared to AD (redistributed to dystrophic neurites, tangles, and within amyloid plaques) brains. Scale bars: 30 μm

In DLPFC sections from subjects (*n* = 3) with no cognitive impairment (NCI) and free from age-related neuropathology, most FADD intensity appeared in the neuronal cell body, especially within nuclei (see merge FADD-NeuN), and neuropil (synapses) (see Fig. 5a). In particular, about 65% of FADD signal intensity was colocalized with NeuN, while 4% of FADD signal intensity was colocalized with syntaxin-1. By contrast, in samples from MAP participants with definite AD (e.g. AD1 and AD2 subjects in Fig. 5a), there was no obvious colocalization between FADD and NeuN labelings in neuronal bodies. Interestingly, FADD appeared to accumulate in dystrophic neurites and tangle-like structures. Notably, the same results were observed for all 3 AD cases, while none of the NCI subjects presented this anomalous distribution of FADD. As these results suggested a possible role for FADD in the mechanisms of pathologic tau deposition, DLPFC sections from subjects with AD were used to study the possible colocalization of FADD with pathological tau (Alz-50) and/or β-amyloid. The results shown in Fig. 5b demonstrated the presence of FADD in tangles and in dystrophic neurites where FADD colocalized with Alz-50 immunoreactivity. FADD was also present in the dystrophic neurites accumulating around neuritic plaques (see Fig. 5b).

### Decreased cortical FADD in APP23 mice with aging

**Fig. 6:**
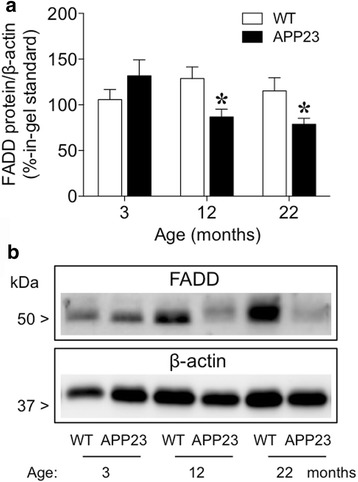
Decreased cortical FADD in APP23 mice with aging. **a** Immunodensity of FADD protein (normalized by β-actin protein content) were quantified by Western blotting in cortical homogenates from APP23 transgenic and wild-type (WT) mice at 3, 12 and 22 months of age. Group of treatment: WT-3 months (*n* = 6), APP23-3 months (*n* = 6), WT-12 months (*n* = 6), APP23-12 months (*n* = 5), WT-22 months (*n* = 6), APP23-22 months (*n* = 5). Columns represent mean values ± SEM per group and expressed as percentage of an in-gel standard. Two-way ANOVA detected an interaction Genotype x Age (F_2,28_ = 4.43, *p* < 0.05). *Post-hoc* multiple comparison *t*-tests revealed significant decreases for adult (12 months old) and aged (22 months old) APP23 mice as compared to age-matched WT controls. *p < 0.05. **b** Representative immunoblots of FADD and β-actin, with one sample per group and age. The molecular masses of the various proteins are indicated in kDa. Full gel images are included in Additional file 2: Figure S2

Given the observed decrease in FADD content in MAP participants with clinical dementia, and the potential association with common AD pathology, we utilized APP23 transgenic mice to further explore the possible role of FADD in a common animal model of AD-like syndrome (i.e., amyloid-β plaques accumulation with age). The results showed, in parallel to the human data, decreased immunodensities of cortical FADD (normalized by β-actin content) in APP23 mice as measured by a two-way ANOVA (interaction Genotype x Age: F_2,28_ = 4.43, *p* = 0.021). *Post-hoc* multiple comparisons via *t*-tests revealed significant decreases for adult (12 months old, *p* = 0.027) and aged (22 months old, *p* = 0.030) APP23 mice as compared to age-matched WT controls (Fig. 6).

## Discussion

Pro-apoptotic FADD was lower in an elderly community-based cohort of subjects with dementia, contrary to the initial prediction of a higher level. Interestingly, lower FADD in the DLPFC of MAP subjects was associated with greater amyloid-β cortical accumulation, reduced synaptic density, microglial activation, lower cognitive function, and higher odds of dementia. In addition, subjects with AD dementia presented an anomalous cortical FADD distribution (i.e., presence in tangles and in dystrophic neurites), compared to NCI subjects (i.e., FADD labeling in neuronal bodies). The redistribution of this adaptor protein into cellular compartments where phosphotau accumulates during the progression of AD may suggest a possible role for FADD in the mechanisms of pathologic tau deposition. Finally, in a common animal model of AD (i.e., APP23 mice), cortical FADD was also decreased, indicating that FADD loss may be caused by age-related amyloid pathology, and suggesting that this multifunctional molecule might be a key component in the amyloid cascade.

Cell death signals mediated by the extrinsic apoptotic pathway are initiated through the interaction of Fas receptor with FADD adaptor, which promotes activation of effector caspases and leads to cell death [22, 62–64]. By contrast, when FADD is phosphorylated and translocated to the nucleus, it mediates anti-apoptotic actions [23, 59]. Thus FADD is a molecule key in controlling cell-fate as it has demonstrated great plasticity in its actions (see [60]). Interestingly, this study found marked reductions in FADD (i.e., pro-apoptotic form), but not p-FADD (i.e., anti-apoptotic form), in the DLPFC of MAP participants displaying clinical dementia and/or a large burden of AD pathology. Similar results were observed in a preliminary postmortem study performed in an independent small subset of patients with AD (n = 5) in which cortical (BA9) FADD was decreased (by about 69%), while p-FADD was unaltered when compared to matched controls (*n* = 4) [65]. In contrast, basal forebrain cholinergic neurons in AD brains expressed high levels of FADD but at the same time did not contain fragmented DNA, a cellular marker of apoptosis [30]. The present results suggest that at the time of death, there were no signs of pro-apoptotic activation mediated by FADD, but a decrease in its content. A possible explanation for FADD decrease could be that initial FADD translocation out of the nucleus (possibly towards the neurites) could have started apoptotic cascades leading to cell death. This massive death of cortical neurons could have further explained the loss of FADD in these subjects. In fact, beyond the amyloid hypothesis biological cascade, the literature suggests a distinct timeline for the increase in biomarker expression (i.e., amyloid β deposits, and hyperphosphorylation of tau) leading to cell death, and the start-point of cognitive decline that culminates in clinical dementia [2]. Another possible explanation would favor the neuroplastic actions of this multifunctional protein suggesting an adaptive response to a prior insult. Interestingly, cognitive decline during aging is due not only to neuronal loss, but is the result of functional changes occurring over time (reviewed in [4]), including synaptic dysfunction (or dysplasticity). According to this model, FADD levels correlate positively with synaptic density markers, such as the SNARE proteins, and negatively with microglia activation, which is thought to be responsible for greater synaptic pruning in AD [66]. Following linear and logistic regression models controlled for multiple confounders, lower FADD levels were associated with increased likelihood of clinical dementia and reduced global cognitive function, partially mediating the effects of amyloid-β accumulation, but not those of phosphotau deposition. Interestingly, these FADD-amyloid-β mediation effects were observed in models where the outcome was clinical dementia (which compared NCI/MCI versus dementia subjects), but not in those predicting cognitive function. Perhaps, this type of interaction between FADD and amyloid-β may play a role in the transition from non-dementia (either NCI or MCI) to dementia.

Immunofluorescence assays were performed to characterize FADD at the cellular and anatomical levels, and to evaluate possible changes in FADD expression patterns in subjects with clinical dementia as compared to NCI controls. Prior studies have suggested that FADD is expressed in human neurons [67, 68] as well as in neuron-enriched cultures from human brain cortex [60]. Other studies have also suggested that FADD is expressed in glial cells (e.g., glioblastoma cell lines; [69]). At the subcellular level, FADD forms are expressed (rodent and human brains) in cytosol and nucleus and to a lesser extent in membranes (e.g., [58]; see revision in [60]). In line with this, the present results showed that NCI subjects accumulated most FADD intensity in the neuronal cell body, especially within nuclei. In contrast, subjects with clinical dementia presented an anomalous FADD distribution, with FADD presence in tangles and in dystrophic neurites, represented by the colocalization observed between FADD and pathological tau (i.e., Alz-50 immunoreacctivity). A report of increased FADD in AD (i.e., within the basal forebrain cholinergic neurons), demonstrated FADD colocalized with phosphorylated tau immunoreactive tangles but not with dense-core amyloid β plaques [30]. These results, in line with ours, suggest a possible role for FADD in the mechanisms of pathologic tau deposition.

Previous observations in transgenic mouse models suggested that cerebral amyloidosis in APP23 mice caused a modest neuronal loss in neocortex at early ages, followed by more neurons with necrotic-apoptotic phenotype in the neocortex at 24 months of age [70]. However, to the best of our knowledge no prior reports evaluated the regulation of apoptotic markers (i.e., FADD) in APP23 mice. In the present study, aged APP23 mice displayed reduced levels of cortical FADD, suggesting FADD loss may be dependent on age-related amyloid pathology. Interestingly, cognitive decline in AD seems to correlate better with neurofibrillary tangles of hyperphosphorylated tau than with amyloid β plaques (see review in [4]). Interestingly, the present results suggest a functional interaction between FADD and pathological tau, but at the same time shows that FADD is sensitive to the accumulation of amyloid β. Therefore, it is reasonable to speculate that FADD might participate in the process of connecting these two classical pathological markers in the progress of clinical dementia, opening room for further studies.

## Conclusions

The present results demonstrate that cortical FADD was decreased in an elderly, community-based cohort subjects with dementia. Interestingly, loss of FADD in the DLPFC was associated with a greater load of amyloid pathology, loss of presynaptic terminal markers, poorer cognitive function and increased risk of dementia. Moreover, subjects with AD presented an anomalous cortical FADD distribution, (i.e., presence in tangles and in dystrophic neurites) as compared to NCI subjects (i.e., FADD labeling in neuronal bodies) suggesting a possible role for FADD in the mechanisms of pathologic tau deposition. Moreover, the decrease in FADD content was consistent with findings in a transgenic mouse model of AD. Overall, the present data suggests FADD as a putative biomarker of the cognitive decline associated with the course of clinical dementia. Future studies should investigate the precise role of this multifunctional adaptor protein within the amyloid cascade, possibly linking plaque-mediated synaptotoxicity and tauopathy in AD.

## Additional files

Additional file 1: Figure S1. Colocalization of FADD and HLA-DR positive (activated) microglia in the DLPFC of neuropathology-free NCI (n = 3) MAP participants. Single-channel (in greys) or merged confocal images correspond to double co-immunolabeled sections with antibodies against FADD (H181, Santa-Cruz, 1:50; magenta) and HLA-DR (clone CR3/43, Dako, 1:100; green). In merged image, colors were arbitrarily assigned to maximize overlap visualization. Overlap panel is an ImageJ-generated bitmap highlighting those pixels where significant colocalization over an unbiased threshold of intensities between the indicated channels was detected in pairwise colocalization analyses. Unlike its neuronal localization pattern, FADD seems absent from the microglial nuclei, and mayor colocalization between these markers appears in activated microglial processes (see yellow arrows). Possibly, FADD microglial inclusions might derive from post-apoptotic neurons. Scale bar: 20 μm. (PDF 292 kb)
Additional file 2: Figure S2. (a) Representative full gel immunoblots of FADD, p-FADD and ß-actin proteins in the DLPFC of MAP participants, with various participants and standard (ST) samples. The red square represents the portion selected for Fig. 3d. (b) Representative full gel immunoblots of FADD and ß-actin proteins in cortical homogenates from APP23 transgenic mice. The red square represents the portion selected for Fig. 5b. The apparent molecular masses of the various proteins were determined by calibrating the blots with prestained molecular weight markers as shown on the left-hand side. (PDF 2893 kb)

## Abbreviations

AD: Alzheimer’s disease
ANCOVA: Analysis of covariance
ANOVA: Analysis of variance
*APOE*: Apolipoprotein E
APP: Amyloid precursor protein
BA: Brodmann’s area
BSA: Bovine serum albumin
CERAD: Consortium to Establish a Registry of Alzheimer’s Disease
CV: Coefficient of variation
DEM: Dementia
DLPFC: Dorsolateral prefrontal cortex
ECL: Enhanced chemiluminescence
FADD: Fas-associated death domain
MAP: Memory and Aging Project
MCI: Mild cognitive impairment
MMSE: Mini mental state examination
NCI: No cognitive impairment
NFTs: Neurofibrillary tangles
NIA: National Institute on Aging
PAGE: Polyacrylamide gel electrophoresis
PBS: Phosphate-buffered saline
PMI: Postmortem interval
SD: Standard deviation
SDS: Sodium dodecyl sulfate
SNAP-25: Synaptosomal-associated protein of 25 kDa
STXBP1: Syntaxin-binding protein-1
VAMP: Vesicle-associated membrane protein
WB: Western blot
WT: Wild-type
