# Supplementary material for: Decreased cortical FADD protein is associated with clinical dementia and cognitive decline in an elderly community sample
Source: Mol Neurodegener. 2017 Mar 20;12:26. doi: 10.1186/s13024-017-0168-x (PMC5360099; doi:10.1186/s13024-017-0168-x)
Supplement: Additional file 1: Figure S1. — Colocalization of FADD and HLA-DR positive (activated) microglia in the DLPFC of neuropathology-free NCI (n = 3) MAP participants. Single-channel (in greys) or merged confocal images correspond to double co-immunolabeled sections with antibodies against FADD (H181, Santa-Cruz, 1:50; magenta) and HLA-DR (clone CR3/43, Dako, 1:100; green). In merged image, colors were arbitrarily assigned to maximize overlap visualization. Overlap panel is an ImageJ-generated bitmap highlighting those pixels where significant colocalization over an unbiased threshold of intensities between the indicated channels was detected in pairwise colocalization analyses. Unlike its neuronal localization pattern, FADD seems absent from the microglial nuclei, and mayor colocalization between these markers appears in activated microglial processes (see yellow arrows). Possibly, FADD microglial inclusions might derive from post-apoptotic neurons. Scale bar: 20 μm. (PDF 292 kb) [file 13024_2017_168_MOESM1_ESM.pdf]

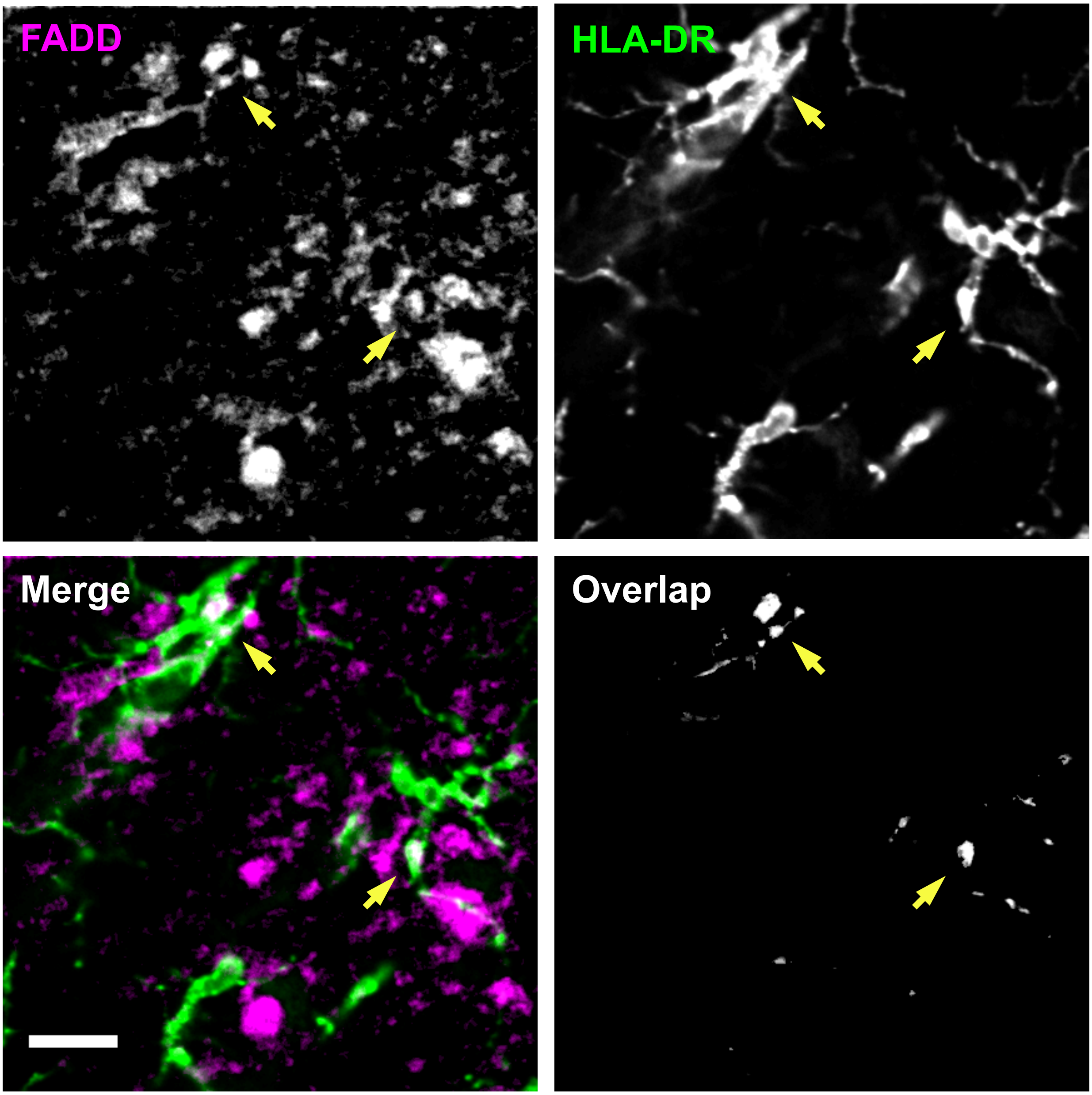

Supplemental Figure S1. Colocalization of FADD and HLA-DR positive (activated) microglia in the DLPFC of neuropathology-free NCI (n = 3) MAP participants. Single-channel (in greys) or merged confocal images correspond to double co-immunolabeled sections with antibodies against FADD (H181, Santa-Cruz, 1:50; magenta) and HLA-DR (clone CR3/43, Dako, 1:100; green). In merged image, colors were arbitrarily assigned to maximize overlap visualization. Overlap panel is an ImageJ-generated bitmap highlighting those pixels where significant colocalization over an unbiased threshold of intensities between the indicated channels was detected in pairwise colocalization analyses. Unlike its neuronal localization pattern, FADD seems absent from the microglial nuclei, and mayor colocalization between these markers appears in activated microglial processes (see yellow arrows). Possibly, FADD microglial inclusions might derive from post-apoptotic neurons. Scale bar: 20 μm.
