# Supplementary material for: Decreased cortical FADD protein is associated with clinical dementia and cognitive decline in an elderly community sample
Source: Mol Neurodegener. 2017 Mar 20;12:26. doi: 10.1186/s13024-017-0168-x (PMC5360099; doi:10.1186/s13024-017-0168-x)
Supplement: Additional file 2: Figure S2. — (a) Representative full gel immunoblots of FADD, p-FADD and ß-actin proteins in the DLPFC of MAP participants, with various participants and standard (ST) samples. The red square represents the portion selected for Fig. 3d. (b) Representative full gel immunoblots of FADD and ß-actin proteins in cortical homogenates from APP23 transgenic mice. The red square represents the portion selected for Fig. 5b. The apparent molecular masses of the various proteins were determined by calibrating the blots with prestained molecular weight markers as shown on the left-hand side. (PDF 2893 kb) [file 13024_2017_168_MOESM2_ESM.pdf]

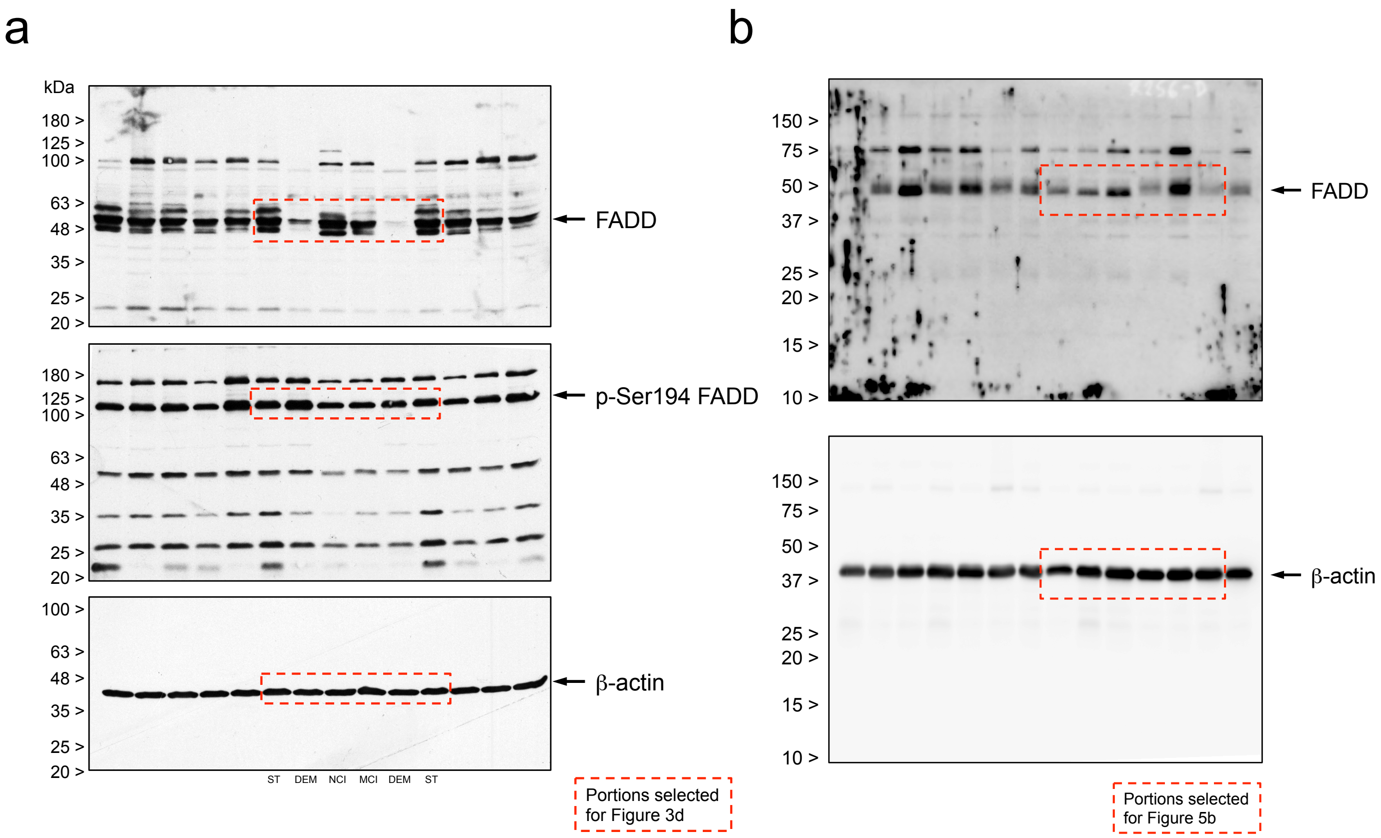

Supplemental Figure S2. (a) Representative full gel immunoblots of FADD, p-FADD and β-actin proteins in the DLPFC of MAP participants, with various participants and standard (ST) samples. The red square represents the portion selected for Figure 3d. (b) Representative full gel immunoblots of FADD and β-actin proteins in cortical homogenates from APP23 transgenic mice. The red square represents the portion selected for Figure 5b. The apparent molecular masses of the various proteins were determined by calibrating the blots with prestained molecular weight markers as shown on the left-hand side .
